# Persistent Expression of Hepatitis C Virus Non-Structural Proteins Leads to Increased Autophagy and Mitochondrial Injury in Human Hepatoma Cells

**DOI:** 10.1371/journal.pone.0028551

**Published:** 2011-12-02

**Authors:** Victor C. Chu, Sayanti Bhattacharya, Ann Nomoto, Jiahui Lin, Syed Kashif Zaidi, Terry D. Oberley, Steven A. Weinman, Salman Azhar, Ting-Ting Huang

**Affiliations:** 1 Department of Neurology and Neurological Sciences, Stanford University, Stanford, California, United States of America; 2 Geriatric Research, Education, and Clinical Center (GRECC), VA Palo Alto Health Care System, Palo Alto, California, United States of America; 3 Department of Pathology and Laboratory Medicine, University of Wisconsin School of Medicine and Public Health, Madison, Wisconsin, United States of America; 4 William S. Middleton Veterans Administration Hospital, Madison, Wisconsin, United States of America; 5 Department of Internal Medicine, University of Kansas Medical Center, Kansas City, Kansas, United States of America; Biological Research Center of the Hungarian Academy of Sciences, Hungary

## Abstract

HCV infection is a major cause of chronic liver disease and liver cancer in the United States. To address the pathogenesis caused by HCV infection, recent studies have focused on the direct cytopathic effects of individual HCV proteins, with the objective of identifying their specific roles in the overall pathogenesis. However, this approach precludes examination of the possible interactions between different HCV proteins and organelles. To obtain a better understanding of the various cytopathic effects of and cellular responses to HCV proteins, we used human hepatoma cells constitutively replicating HCV RNA encoding either the full-length polyprotein or the non-structural proteins, or cells constitutively expressing the structural protein core, to model the state of persistent HCV infection and examined the combination of various HCV proteins in cellular pathogenesis. Increased reactive oxygen species (ROS) generation in the mitochondria, mitochondrial injury and degeneration, and increased lipid accumulation were common among all HCV protein-expressing cells regardless of whether they expressed the structural or non-structural proteins. Expression of the non-structural proteins also led to increased oxidative stress in the cytosol, membrane blebbing in the endoplasmic reticulum, and accumulation of autophagocytic vacuoles. Alterations of cellular redox state, on the other hand, significantly changed the level of autophagy, suggesting a direct link between oxidative stress and HCV-mediated activation of autophagy. With the wide-spread cytopathic effects, cells with the full-length HCV polyprotein showed a modest antioxidant response and exhibited a significant increase in population doubling time and a concomitant decrease in cyclin D1. In contrast, cells expressing the non-structural proteins were able to launch a vigorous antioxidant response with up-regulation of antioxidant enzymes. The population doubling time and cyclin D1 level were also comparable to that of control cells. Finally, the cytopathic effects of core protein appeared to focus on the mitochondria without remarkable disturbances in the cytosol.

## Introduction

Hepatitis C virus (HCV) is an enveloped, positive, single-stranded RNA virus in the family of *Flaviviridae* [1]. The linear, non-segmented HCV genome of 9.6 kb encodes a polyprotein that undergoes post-translational cleavage by cellular and viral proteases to yield at least 10 mature proteins [2]–[4]. HCV infection is a major cause of chronic liver disease and is the major cause of liver cancer in the United States. HCV produces a chronic infection in 50–80% of infected patients; among them, roughly 20% will eventually develop liver cirrhosis. It is widely accepted that insufficient host immune response in eliminating HCV leads to persistent infection and the eventual development of liver diseases [4]–[6]. Interferon-α and ribavirin treatments have been prescribed either to stimulate immune response for clearance of viruses or to disrupt viral replication. However, high toxicity and low efficacy toward the two most prevalent HCV subtypes, 1a and 1b, in the US has been a barrier to effective eradication of persistent HCV infections [7].

To address the pathogenesis caused by HCV infection, recent studies have begun to focus on direct cytopathic effects. HCV proteins associate with different subcellular structures, including mitochondria, endoplasmic reticulum (ER), and lipid droplets, to facilitate replication and assembly of viral particles [2]. These associations lead to alterations of the integrity and functions of organelles. HCV-mediated oxidative stress is commonly observed and is achieved by increasing reactive oxygen and nitrogen species (ROS and RNS) or by altering cellular antioxidant capacities [8]–[11]. In particular, HCV core proteins are shown to be closely associated with the mitochondria and cause increases in ROS and RNS production and lipid peroxidation [11]–[14], reduction in GSH and NADPH concentrations, reduction in mitochondrial complex I activities, and increase in mitochondrial Ca^+2^ uptake, which ultimately disrupts mitochondrial membrane permeability and leads to mitochondrial dysfunction [14], [15]. HCV non-structural proteins have also been implicated in disturbing the redox balance and altering antioxidant enzyme levels [16], [17]. Specifically, NS5A is shown to up-regulate Mn superoxide dismutase (MnSOD) through AP1 transcription factor in the p38 MAPK and JNK signaling pathways [18], [19]. Additional studies showed the involvement of NS5A in ER stress and disturbance of intracellular Ca^+2^ homeostasis, which leads to increased mitochondrial ROS production and altered mitochondrial function [18], [20]. Because of the relationship between chronic HCV infection and the development of hepatocellular carcinoma, studies have also been carried out to identify HCV proteins that may be responsible for the hepatocarcinogenesis. For example, the HCV core protein has been shown to promote immortalization of primary human hepatocytes [21], whereas the non-structural proteins NS3 and NS4B have been shown to transform NIH 3T3 cells either individually or in combination with Ha-ras [22], [23].

Most studies have focused on the direct cytopathic effects of individual HCV proteins, with the objective of identifying their specific roles in the overall pathogenesis. However, this approach precludes examination of the possible interactions between different HCV proteins and organelles. We hypothesize that different components of HCV polyprotein, depending on their subcellular distributions, cause different types of cytopathic effects and elicit different types of responses in different subcellular compartments. To obtain a better understanding of the various cytopathic effects of and cellular responses to HCV proteins, we used hepatoma cells constitutively expressing the HCV genome-length replicon, the subgenomic replicon, or the core protein to model the state of persistent HCV infection and took a comparative approach to dissecting the role of various HCV proteins in cellular pathogenesis in this study.

## Results

### Expression and subcellular localization of HCV proteins

**Figure 1:**
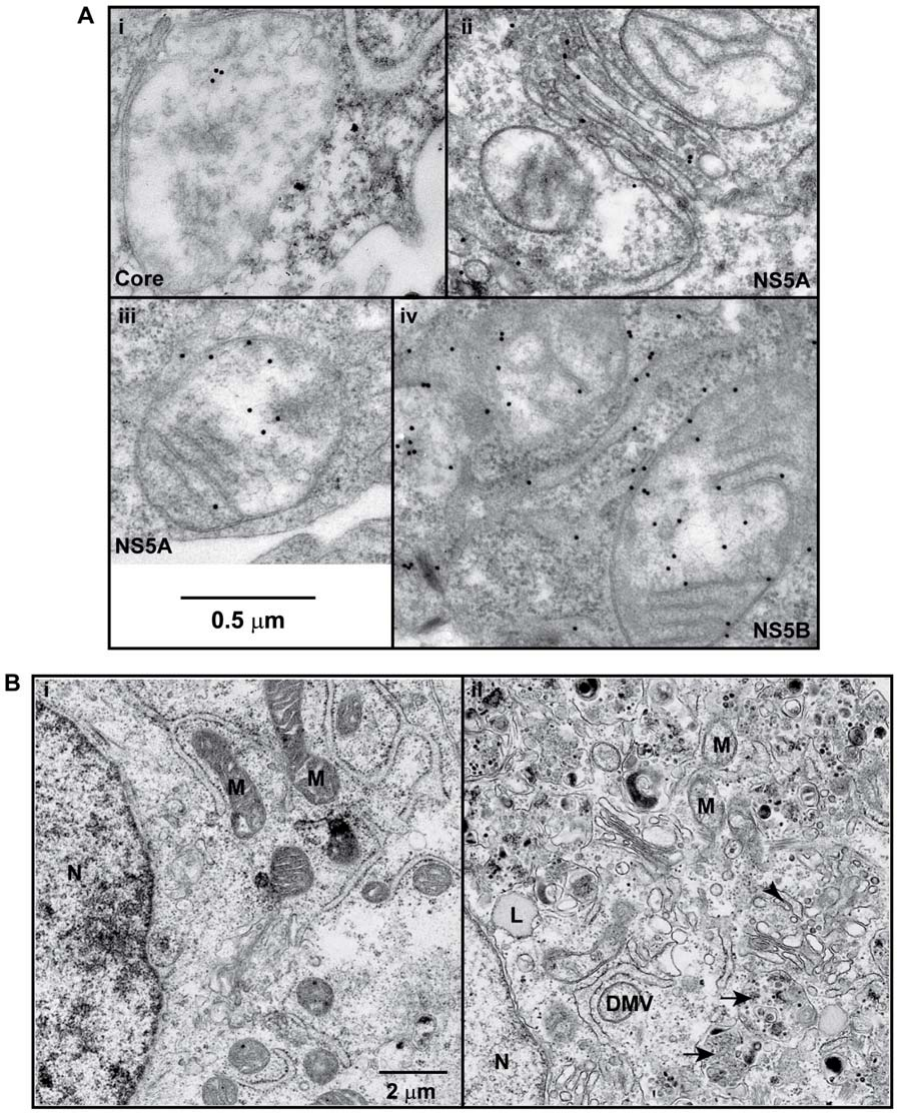
Immunogold localization of HCV proteins and alteration of ultrastructure in cells expressing HCV proteins. A, immunogold EM analyses. (i), Core proteins on the inner membrane of mitochondria; (ii), NS5A in the Golgi; (iii), NS5A in the matrix and inner membrane of mitochondria; (iv), NS5B in the mitochondria (binding to the outer membrane, inner membrane and the matrix) and rough endoplasmic reticulum (RER). Gold particles  = 15 nm. B, transmission electron microscopy analysis of ultrastructure. (i) Huh7 cells with compact, dense mitochondria (M) and RER; (ii) a representative image from HCV genome-length replicon cells with enlarged mitochondria (M) with focal loss of cristae, accumulation of lipid droplets (L), focally dilated RER (arrow head), and the presence of autophagocytic vacuoles (arrow) and double membrane vesicles (DMV).

Based on the SEAP activities measured from multiple sampling, the protein expression levels of HCV genome-length and subgenomic replicons were comparable (Figure S1A). Core protein expression levels, on the other hand, suggested that Core-on cells produced higher levels of core protein than the genome-length replicon cells (Figure S1B). To identify the subcellular location of HCV proteins in genome-length replicon, subgenomic replicon, and Core-on cells, immunogold EM was carried out with antibodies against core, NS5A, and NS5B proteins. The majority of core, NS5A, and NS5B proteins were located in the mitochondria and the ER with minor differences in their distribution within these organelles (Figure 1A and Table S1). Positive signals were also observed in the nucleus, lipid droplets, and the Golgi. NS5A signals were observed in autophagocytic vacuoles in 25% of the electron micrographs examined, with an average signal intensity of 3–6 gold particles per autophagocytic vacuole (Table S1).

### Ultrastructural changes

**Figure 2:**
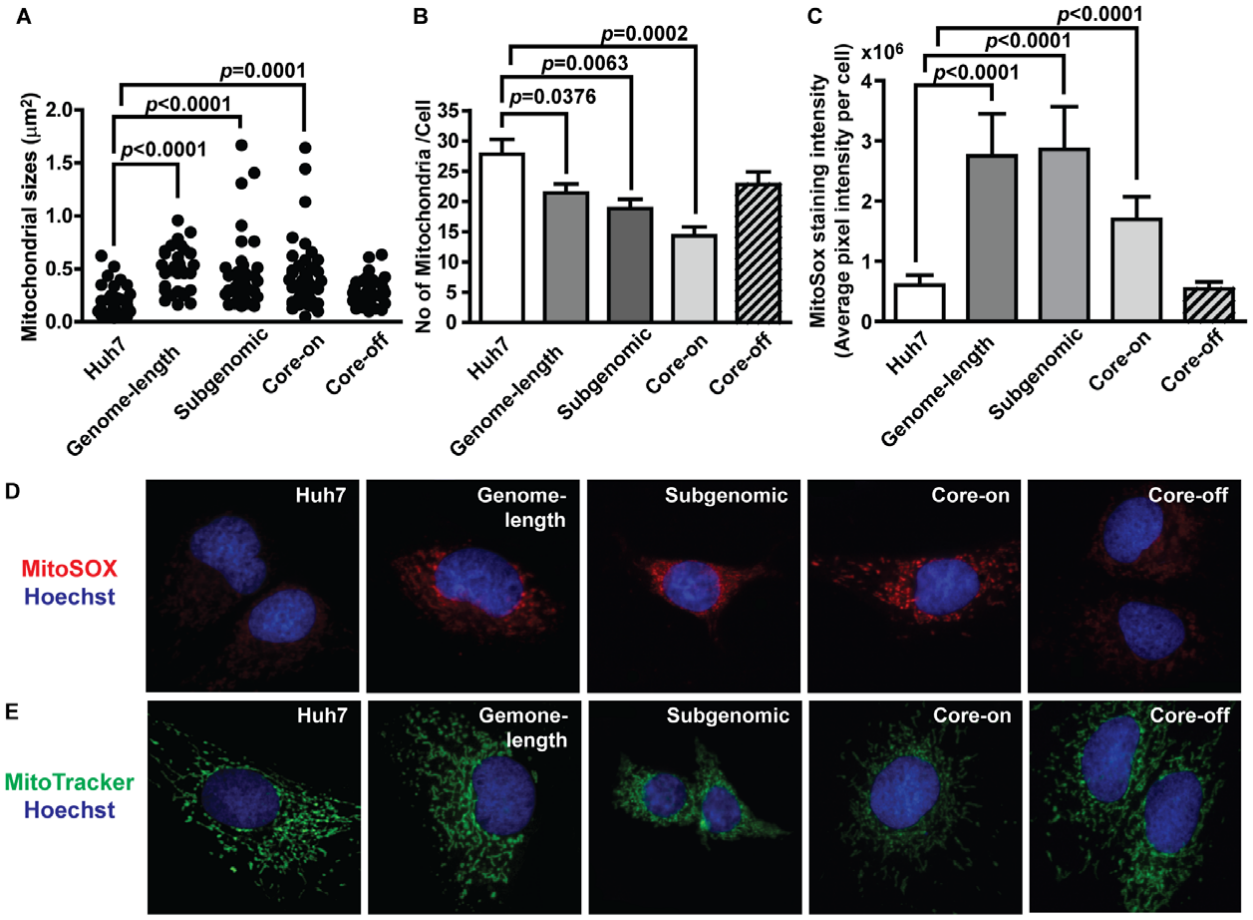
Mitochondrial defects associated with the presence of HCV proteins. A, mitochondrial sizes determined from electron micrographs from different HCV protein-expressing Huh7 cells; B, average number of mitochondria per cell. C, MitoSOX staining intensity quantified as average pixel intensity per cell; D, images of MitoSOX staining showing increased ROS production in the mitochondria of genome-length replicon, subgenomic replicon, and Core-on cells. E, MitoTracker Green stain showing the density and distribution of mitochondria. Nuclei are stained with Hoechst. Data in B and C are presented as mean ± SEM. A minimum of 40 cells each were analyzed.

**Figure 3:**
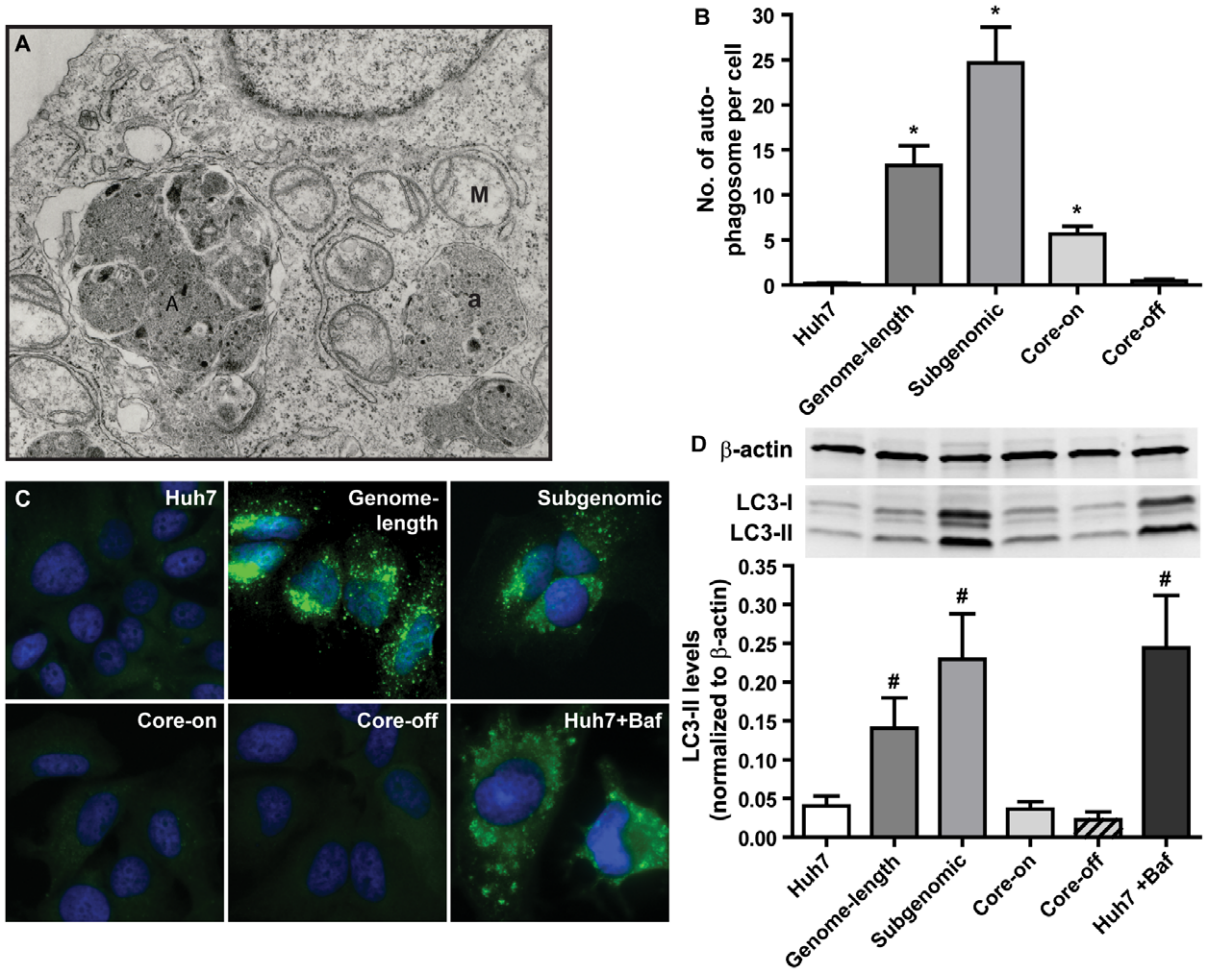
Enhanced steady-state autophagy in genome-length and subgenomic replicon cells. A, a representative EM image from subgenomic replicon cells showing the presence of autophagocytic vacuoles (A) and autophagocytic vesicles (a). Enlarged mitochondria (M) are also evident in this image. B, autophagosome counts per cell. A minimum of 12 cells each were analyzed. Data are presented as mean ± SEM. *, *p*<0.0001. C, immunocytochemical staining of LC3. The pixilated staining pattern of LC3, which is an indication of autophagosome formation and maturation, is consistently observed in genome-length and subgenomic replicon cells. Huh7 treated with Bafilomycin (Baf) was used as a positive control. D, western blot quantification of LC3-II. The upper panel shows a representative western blot result and the lower panel shows the quantification of LC3-II levels. Total LC3 levels (LC3-I + LC3-II) were also significantly increased in subgenomic replicon cells (p = 0.0028) and Huh7 cells treated with Bafilomycin (p = 0.0278). Data are presented as mean ± SEM of four independent experiments. LC3 levels were normalized to that of β-actin. #, *p*<0.05.

To identify ultrastructural changes caused by the presence of HCV proteins, electron microscopic analyses were carried out. The study results revealed mitochondrial injury as a common defect among genome-length replicon, subgenomic replicon, and Core-on cells with enlarged mitochondria and focal loss of cristae (Figure 1B). Consistent with this observation, measurement of the cross sectional area of mitochondria directly from electron micrographs showed a significant increase in mitochondrial sizes (Figure 2A). The data indicates mitochondrial degeneration and the possible loss of mitochondria. Consequently, the number of mitochondria per cell was significantly reduced (Figure 2B). In addition to mitochondrial defects, accumulation of lipid droplets was common among all three HCV protein-expressing cell lines (Figure 1B and Figure S2). Genome-length and subgenomic replicon cells also showed focally dilated rough endoplasmic reticulum (RER) and prominent presence of autophagocytic vacuoles (Figures 1B and 3A).

### Increased oxidative stress in the mitochondria and cytosol

Increased oxidative damage is the likely culprit for the observed mitochondrial degeneration and reduced mitochondrial number in HCV protein-expressing cells. To determine the extent of ROS generation in the mitochondria, MitoSOX was used for live cell staining. The results showed a significant increase in ROS production in the mitochondria across all three cell lines (Figures 2C and 2D). After adjusting to the average number of mitochondria per cell, the level of MitoSOX intensity was similar among genome-length replicon, subgenomic replicon, and Core-on cells. Cytosolic and mitochondrial aconitases (ACO1 and ACO2) are very sensitive to inactivation by reactive oxygen and nitrogen species, and reduction in aconitase protein levels and enzyme activities have been used as indicators for increased oxidative stress in their respective subcellular compartments [24], [25]. Our studies showed that ACO1 existed at low levels, whereas ACO2 was barely detectable by western blot analysis, in Huh7 and HCV protein-expressing cells. ACO1 protein level was reduced by 18% in cells with the genome-length replicon (Figure S3A), and total aconitase activity was reduced by 5–23% (Figure S3B) in genome-length and subgenomic replicon cells. The reduction in total aconitase activity most likely reflected increased oxidative stress in the cytosol because the majority of aconitase in Huh7 cells was in cytosolic form.

### Increased steady state autophagy in cells expressing full length and non-structural HCV proteins

From ultrastructure analyses, we consistently observed the presence of autophagocytic vacuoles and primary autophagocytic vesicles (Figures 1B, 3A, and 3B) in genome-length replicon and subgenomic replicon cells and, to a lesser extent, in Core-on cells. To confirm the initial observation, immunocytochemical and western blot analyses were carried out to determine the status of LC3, which is an integral part of the autophagosome membrane. LC3-positive punctuate structures in the cytoplasm were prominent in genome-length replicon and subgenomic replicon cells (Figure 3C), but they were only present at a very low level in Core-on cells. Consistent with this result, western blot analyses showed a marked increase of LC3-I and LC3-II in genome-length replicon and subgenomic replicon cells (Figure 3D). However, the ratios between LC3-I and II were not changed. In contrast to the prominent accumulation of autophagocytic vacuoles in genome-length and subgenomic replicon cells, autophagosomes were not detected in HCV transgenic mice expressing the full-length HCV polyprotein (data not shown).

### Redox status affects HCV-mediated activation of autophagy

**Figure 4:**
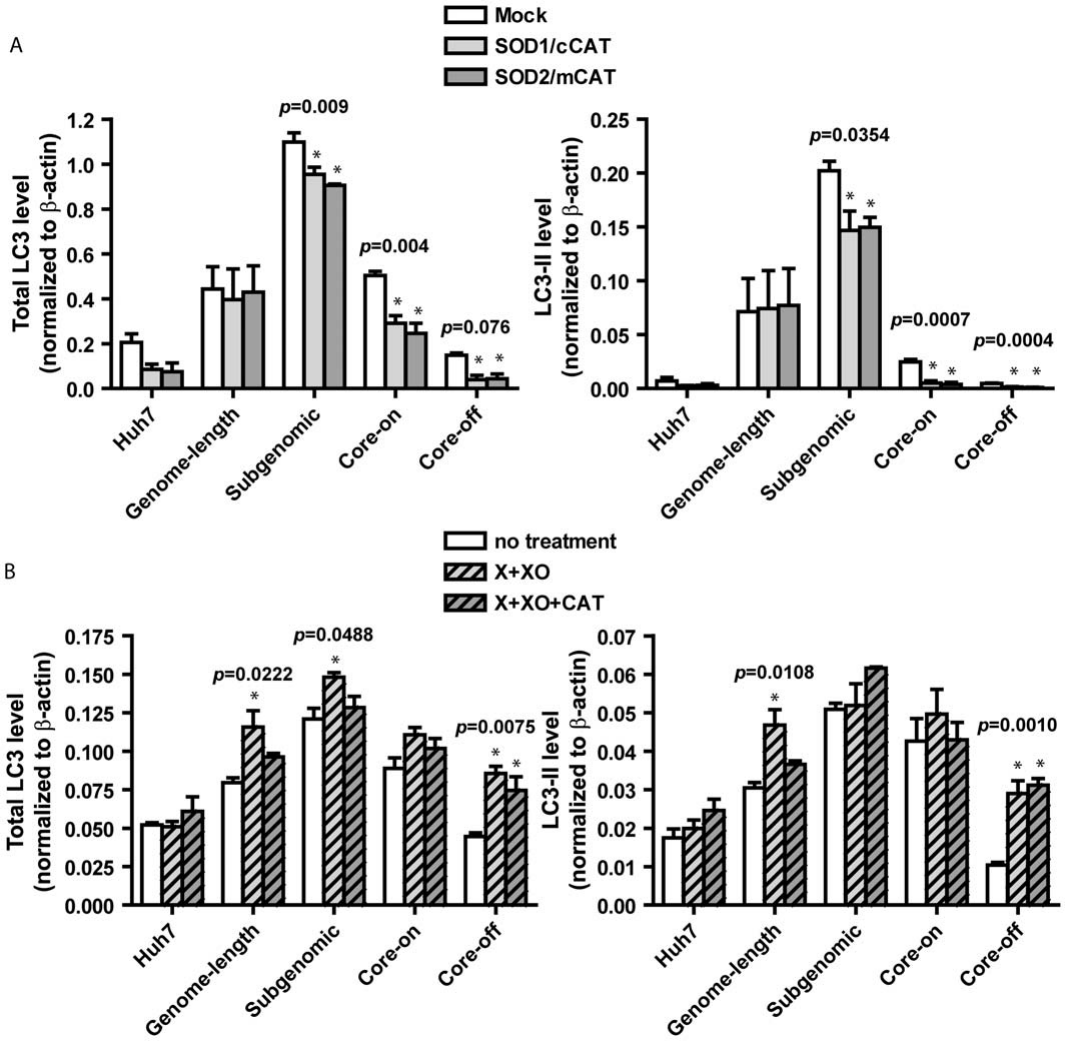
Redox state affects HCV-mediated autophagy. Total LC3 (LC3-I + LC3-II) and LC3-II levels were quantified by western blot analyses. A, Dual expression vectors for increasing cytosolic (SOD1/cCAT) or mitochondrial (SOD2/mCAT) antioxidant expression were used, and mock-transfected cells served as controls. Cells were analyzed 36 hrs after the initial transfection. B, Xanthine (X) and xanthine oxidase (XO) were used to generate ROS to increased oxidative stress. To remove hydrogen peroxide and leaving only superoxide in the X/XO system, catalase was added to the reaction (X/XO/CAT). Cells were treated for 72 hrs. Data are presented as mean ± SEM of three independent experiments. LC3 levels were normalized to that of β-actin. Significant results from one-way ANOVA are shown. *, *p*<0.05 when compared to mock or no treatment controls.

To determine if oxidative stress played a role in HCV-mediated activation of autophagy, cellular redox state was altered by either enhancing the cellular antioxidant capacity through dual overexpression of superoxide dismutase (SOD) and catalase (CAT), or increasing cellular oxidative stress by xanthine/xanthine oxidase (X/XO) treatment. Changes in total LC3 and LC3-II levels were used as indicators of autophagy. Significant reduction of total LC3 and LC3-II levels was observed in subgenomic replicon, Core-on, and Core-off cells with overexpression of CuZnSOD/cytosolic catalase (SOD1/cCAT) or MnSOD/mitochondrial catalase (SOD2/mCAT) (Figure 4A). Although similar trends were observed in Huh7 cells, the extent of reduction did not reach a significant level. Despite comparable levels of CuZnSOD, MnSOD, and catalase expression (Figure S4), LC3 levels were not altered in genome-length replicon cells. In contrast, X/XO treatment significantly increased total LC3 levels in genomic replicon, subgenomic replicon, and Core-off cells (Figure 4B, left panel). However, LC3-II levels were only significantly increased in genomic replicon and Core-off cells (Figure 4B, right panel). No significant change in LC3-II level was observed in Huh7, subgenomic replicon, and Core-on cells. Furthermore, addition of CAT to the X/XO treatment diminished the increase of total LC3 in genome-length and subgenomic cells (Figure 4B). The data suggest that the enhanced autophagy from X/XO treatment is mediated through H_2_O_2_ in genome-length and subgenomic replicon cells.

### Upregulation of antioxidant enzymes in cells expressing HCV subgenomic replicon

**Figure 5:**
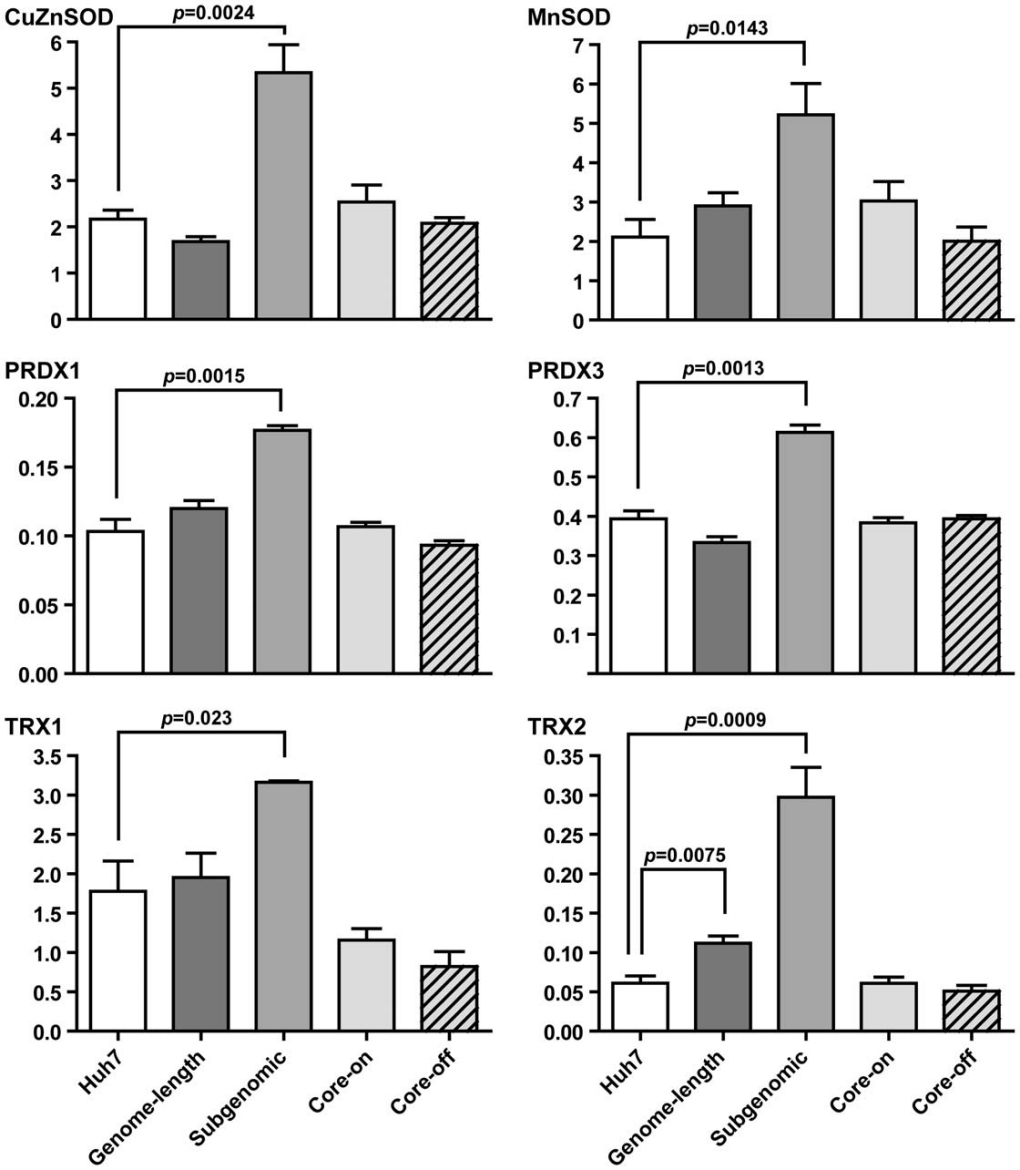
Antioxidant profiles in HCV protein-expressing cells. Protein levels of antioxidant enzymes in the cytosol (left panels) and mitochondria (right panels) were determined by western blot analyses. Data are presented as mean ± SEM of three (PRDX and TRX) or four (SOD) independent experiments. All protein levels were normalized to that of β-actin.

To find out whether increased mitochondrial stress and ER stress led to changes in antioxidant profiles, western blot analyses were carried out to determine the protein levels of CuZnSOD, MnSOD, peroxiredoxin 1 (PRDX1), peroxiredoxin 3 (PRDX3), thioredoxin 1 (TRX1), and thioredoxin 2 (TRX2). Among them, CuZnSOD, PRDX1, and TRX1 are cytosolic proteins, and MnSOD, PRDX3, and TRX2 are mitochondrial proteins. The sulfonylated peroxiredoxins (PRDX-SO3), which are the end products of irreversible oxidation of the active site cysteine in peroxiredoxins, were also monitored and served as indicators for the redox state in HCV protein-expressing cells (Figure S5C). Significant increases in the protein levels of CuZnSOD, MnSOD, PRDX1, PRDX3, TRX1, and TRX2 were consistently observed in the subgenomic replicon cells, and the extent of increase ranges from 1.5- to 4.9-fold (Figure 5). The genome-length replicon cells showed a modest (1.8-fold) but significant increase in TRX2, and a marginal decrease in CuZnSOD (p = 0.0734) and PRDX3 (p = 0.0739) (Figure 5). No remarkable changes were observed between Core-on and Core-off cells. The ratios of PRDX1-SO3/PRDX1 and PRDX3-SO3/PRDX3 were also used to gauge the redox state in the cytosol and the mitochondria, respectively, and no significant changes were observed across all cell lines analyzed (Figure S5A and S5B).

### Population doubling time is increased in cells expressing the genome-length replicon

**Figure 6:**
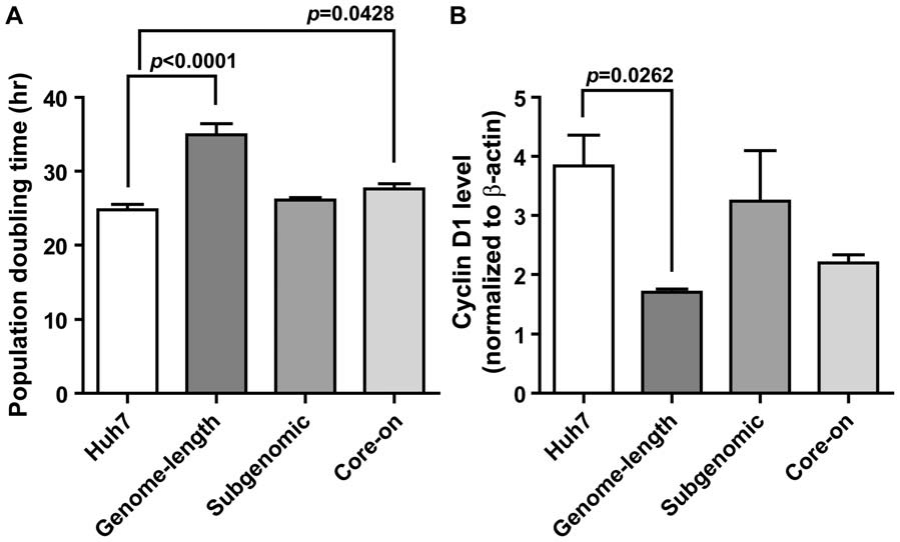
Population doubling time (A) and cyclin D1 level (B) in HCV protein-expressing Huh7 cells. Data are presented as mean ± SEM of 4–5 (population doubling time) or three (cyclin D1) independent experiments.

To determine if ultrastructural changes and increased mitochondrial ROS production affect cell proliferation and survival, population doubling time was determined during the exponential phase of cell growth. Genome-length replicon and Core-on cells showed a 41% and 11% increase in population doubling time, respectively, without a significant increase in cell death during the 72-hr period when cell number increase was monitored (Figure 6A). On the other hand, subgenomic replicon cells had a comparable population doubling time to that of Huh7 and Core-off cells. To determine if cell cycle regulation is affected in HCV protein-expressing cells, in-cell westerns were carried out to determine total cyclin D1 levels. Consistent with increased population doubling time in genome-length replicon cells, cyclin D1 level was decreased (Figure 6B). There was also a trend in decreased cyclin D1 levels in Core-on cells.

## Discussion

In this study, we showed that the most dominant subcellular location for HCV core, NS5A, and NS5B proteins was in the mitochondria, followed by the ER and the Golgi. Consequently, expression of HCV core or non-structural proteins led to increased ROS generation in the mitochondria, which most likely contributed to mitochondrial injury and degeneration. Expression of non-structural proteins also led to membrane blebbing in the ER and accumulation of autophagocytic vacuoles. Despite these changes, only cells with the subgenomic replicon (i.e. cells expressing the non-structural proteins) showed consistent up-regulation of mitochondrial and cytosolic antioxidant enzymes. Cells with the genome-length replicon, on the other hand, did not have a robust antioxidant response and showed prolonged population doubling time and a decrease in cyclin D1 expression. Apart from the shared mitochondrial phenotype, increased lipid accumulation was also common among all three HCV protein-expressing cell lines.

Subcellular locations of HCV proteins have provided important clues to the site of viral genome replication and assembly, as well as the cytopathic effects caused by HCV infection. The ultrastructure study with immunogold labeling showed a distinct localization of core, NS5A, and NS5B proteins in the mitochondria and the ER. Significant core, NS5A, and NS5B distributions were observed in other subcellular compartments, including the Golgi apparatus, the nucleus, and lipid droplets. The study results are in general agreement with previously published data [26]–[31]. However, the inner membrane location of core protein was unexpected because previous studies using proteinase K digestion suggested association of core protein with the mitochondrial outer membrane and the mitochondrial-associated membrane compartment [15], [28]. The discrepancy could be due to the compromised mitochondrial membrane integrity in genome-length replicon and Core-on cells, or due to artifacts from sample preparations for EM analysis. The association of core protein with lipid droplets also appeared to be lower than previous study results, and the discrepancy could be due to differences in the antibodies used.

A significant number of studies have focused on the role of HCV core protein in mitochondrial dysfunction [11], [14], [15], [20], [26], [32]. In this study, cells expressing only the non-structural proteins were also shown to have suffered from mitochondrial damage, and the extent of damage, in terms of mitochondrial size and number, was comparable to that of cells expressing the full-length polyprotein or core protein (Figures 2A and 2B). In addition, the level of ROS production in the mitochondria, as detected by MitoSOX, was also comparable among the three HCV protein-expressing cell lines after adjusting for total mitochondria (Figures 2C and 2D). The data suggest that multiple HCV proteins are capable of entering the mitochondria and cause increased oxidative stress, although the underlying mechanism may not be the same for each protein. The core protein-expressing cells had the lowest number of mitochondria per cell (Figure 2B). The data implies that Core-on cells may suffer from the most severe mitochondrial loss among the three HCV protein-expressing cell lines. Whether this is due to the higher level of core protein expression in Core-on cells (Figure S1A) will need to be examined with other approaches.

Besides increased ROS production in the mitochondria of all three cell lines analyzed, our data on aconitase protein levels and enzyme activities also suggested an increased ROS production in the cytosol in genome-length and subgenomic replicon cells. The reduction in ACO1 protein level in genome-length replicon cells is reminiscent to that observed in CuZnSOD deficient mice [25], in which heightened state of oxidative stress in the cytosol may lead to increased degradation of irreversibly inactivated aconitase. Subgenomic replicon cells had reduced aconitase activity without a significant reduction in the protein level (Figures S3A and S3B). The data suggests that ACO1 may be reversibly inactivated due to increased oxidative stress in the cytosol. Taken together, our study results suggest that HCV non-structural proteins are capable of causing increased oxidative stress in both the mitochondrial and the cytosolic compartments, whereas the effects of core protein is mainly limited to the mitochondria.

Cells respond to a variety of internal stress or cellular damages by forming autophagosomes to degrade damaged components and to recycle usable elements for future biosynthesis [33]. However, some RNA viruses, most notably, poliovirus, dengue virus, mouse hepatitis virus, and foot-and-mouth disease virus develop the abilities to overcome such cellular defense by hijacking the autophagocytic pathway to facilitate viral replication [34]–[37]. Multiple studies in recent years have shown that HCV infection of the human hepatoma cell line Huh7 and immortalized human hepatocytes induce autophagocytic vacuoles [38], [39] and that autophagy machinery is required for the initiation of HCV replication [40] and the production of infectious particles [41]. HCV induces the accumulation of autophagosomes via activation of the unfolded protein response pathway without enhancing autophagocytic protein degradation [38], [42]. Consequently, inhibition of autophagy inhibits HCV replication [41]. Although most studies are carried out with HCV genotype 2a, it is now well accepted that autophagy probably also occurs with infection of other HCV genotypes. What is not clear from these studies is whether enhanced autophagy persists during the chronic phase of HCV infection, and if and to what extent oxidative stress contributes to HCV-mediated activation of autophagy. In this study, we observed the accumulation of autophagocytic vacuoles and up-regulation of the autophagosome marker, LC3, in the genome-length and subgenomic replicon cells (Figures 3B–3D). The data suggests that HCV non-structural proteins play a role in inducing autophagy, possibly through ER stress (Figure 1B), mitochondrial damage (Figures 1B and 2A), and induction of oxidative stress (Figures 2D and 4B). Core-on cells, on the other hand, had a slight increase in the number of autophagocytic vacuoles without a significant increase in LC3-II or total LC3 levels (Figures 3B-3D).

HCV-mediated oxidative stress was likely a contributor in the activation of autophagy because enhanced antioxidant capacity significantly reduced total LC3 and LC3-II levels (Figure 4A), whereas increased oxidative stress led to a further increase in total LC3 and LC3-II (Figure 4B). ROS generated in the cytosol and mitochondria was equally capable of enhancing autophagy because increased antioxidant capacity in either compartment achieved comparable levels of reduction. In addition, H_2_O_2_ appeared to be the main culprit in ROS-mediated activation of autophagy within this experimental system since addition of catalase effectively suppressed X/XO-mediated activation of autophagy (Figure 4B). Despite up-regulation of multiple antioxidant enzymes in subgenomic replicon cells (Figure 5), these cells responded to further increase in antioxidant enzymes with reduced autophagy. It is important to note that although the antioxidant enzymes clearly can modulate the effects of viral proteins, they only serve to reduce the LC3 increase by ∼26% in subgenomic replicon cells. The data suggest either that other mechanisms, such as ER stress, are important as well or that the antioxidant effect is incomplete. Overexpression of antioxidant enzymes also led to a reduction of autophagy in Core-on and Core-off cells. Antioxidant enzyme overexpression in cells with genome-length replicon, on the other hand, failed to reduce the level of autophagy even though the enhanced antioxidant capacity from SOD/CAT expression vectors was comparable to that of other cells (Figure S4). In contrast, increased oxidative stress effectively increased autophagy in genome-length replicon cells (Figure 4B). The data suggest that oxidative stress may not play a significant role in the basal level of autophagy in genome-length replicon cells; however, the cells are sensitive to additional oxidative stress and are capable of accumulating more autophagosomes under conditions of increased oxidative stress. Although total LC3 levels were increased in X/XO-treated subgenomic replicon cells, LC3-II levels were not changed with the added oxidative stress (Figure 4B). It is possible that the conversion of LC3-I to LC3-II was already at the maximum level in subgenomic replicon cells, and additional oxidative stress was not able to enhance the reaction further.

NS5A and, to a lesser extent, NS5B and the core protein have been detected in autophagocytic vacuoles (Table S1). Whether the NS5A localization in the autophagosome observed in our study is a cellular defense mechanism elicited to degrade foreign antigens or a viral mediated self-preserving mechanism to enable viral replication remains to be determined. HCV transgenic mice expressing the full-length HCV polyprotein were also examined for the accumulation of autophagocytic vacuoles in the liver. However, no evidence of enhanced autophagy was detected. It is possible that additional factors are needed to cause the accumulation of autophagosomes *in vivo* or that very low viral protein expression levels in the transgenic mice were not sufficient to induce these changes. A previous study using the same line of transgenic mice [43] showed that long-term iron overload was necessary to induce the accumulation of autophagosomes. Since iron overload increases oxidative stress in the liver, the result is consistent with our finding in HCV protein-expressing cells in the relationship between oxidative stress and autophagy.

Huh7 cells with the subgenomic replicon showed significant up-regulation of multiple antioxidant enzymes that belonged to the cytosolic and mitochondrial compartments (Figure 5). Despite the up-regulation of multiple antioxidant enzymes, the ratios of oxidized (Sulfonic form) to total peroxiredoxin 1 and 3 in the subgenomic replicon cells remained the same as that in Huh7 cells (Figure S5), suggesting that the overall redox environment was still more oxidizing. This conclusion was also supported by the increased MitoSOX staining and reduced aconitase activities (Figures 2C and 2D and Figure S3B). Whether the up-regulation of multiple antioxidant enzymes in the subgenomic replicon cells is a direct response to the presence of HCV non-structural proteins or is merely a clonal variation will need to be deciphered with additional studies in the future. However, it is worth noting that previous studies with different HCV subgenomic replicon-expressing cells also showed a similar up-regulation in various antioxidant enzymes [17], [44]. Huh7 cells with the genome-length replicon had a 1.8-fold increase in the mitochondrial form of thioredoxin (TRX2), but a 15% reduction of its upstream enzyme PRDX3 (Figure 5). The net result was a slightly higher ratio of PRDX3-SO3 to total PRDX3; however, the increase was not significantly higher than that of Huh7 cells (Figure S5B). The expression level of non-structural proteins should be comparable between the genome-length and subgenomic replicon cells based on the SEAP reporter assay (Figure S1A) and published results [45]. Therefore, the lack of antioxidant response in cells with the genome-length replicon was probably not due to a difference in the expression of non-structural proteins; but rather, it could be due to the additional cytopathic effects from core, E1, E2, and p7 proteins that counteract the antioxidant enzyme response induced by non-structural proteins. Consequently, the genome-length replicon cells had a prolonged population doubling time and reduced cyclin D1 expression (Figure 6), which suggested delayed cell cycle progression. In contrast, Core-on cells showed no changes in any of the antioxidant enzymes examined.

**Figure 7:**
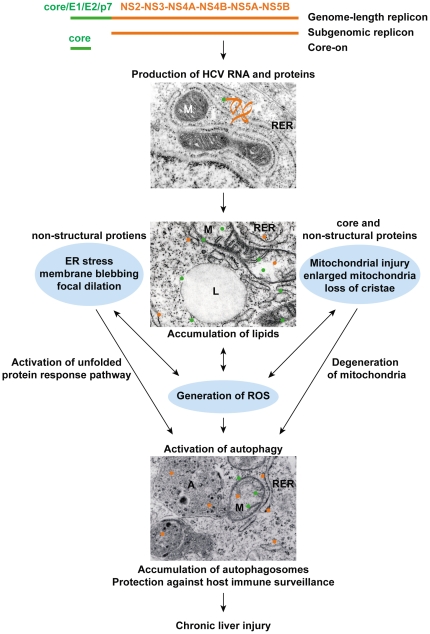
Diagrammatic presentation of the cytopathic effects caused by various HCV proteins. Replication of HCV RNA and production of HCV structural (core, E1, E2, and p7) and non-structural proteins (NS2, NS3, NS4A, NS4B, NS5A, and NS5B) in the rough ER and the subsequent partition of HCV proteins to different subcellular compartments lead to ER stress, mitochondrial injury, and the production of ROS. These cytopathic effects lead to activation of autophagy without a concomitant increase in protein degradation. Consequently, autophagosomes accumulate in HCV infected cells. EM photo with normal mitochondria (M) and rough ER (RER) was taken from healthy Huh7 cells; EM photos with enlarged mitochondria, ER blebbing, lipid droplet (L), and autophagosomes (A) were taken from genome-length and subgenomic replicon cells. The HCV genetic materials contained in genome-length replicon, subgenomic replicon, and Core-on cells are depicted at the top of the diagram.

In summary, our studies using Huh7 cells expressing different parts of the HCV polyprotein suggest that the presence of the full-length HCV polyprotein leads to the most severe cellular damage, including generation of ROS, accumulation of lipids, ER stress, mitochondrial injury and degeneration, accumulation of autophagosomes, and prolonged population doubling time. Despite the elevated levels of ROS, these cells failed to mount a robust antioxidant response. Huh7 cells with only the non-structural proteins, on the other hand, have the same cytopathic changes as that observed in the genome-length replicon cells, but the cells showed a robust antioxidant response even though the response was not sufficient to suppress HCV-mediated ROS production. Our data suggest that HCV-mediated ER stress, mitochondrial injury, and oxidative stress form the three arms of mediators for the activation of autophagy (Figure 7). Their effects are additive and inter-related. Without a concomitant increase in downstream protein degradation, autophagosomes accumulate and provide a sanctuary for HCV replication and protection from host immune surveillance [42]. The process likely helps to sustain a low level of viral production, which perpetuates chronic HCV infection and chronic liver injury (Figure 7). In addition to activation of autophagy, HCV-mediated ER stress can lead to imbalance in Ca homeostasis, which further contributes to cellular oxidative stress [14], [15], [46], [47]. Mitochondrial injury can lead to metabolic deficits and further exacerbates cell injury and dysfunction. Oxidative stress can cause increased DNA damage and mutation, inactivation of redox-sensitive proteins, and activation of redox sensitive signaling pathways such as MAPK and AP-1 [48]–[50]. These cytopathic effects work in concert in the course of chronic HCV infection to promote cell death, hepatocyte turnover, alteration of the liver microenvironment, and ultimately cell transformation and tumorigenesis.

## Materials and Methods

### Ethics Statement

All animal procedures were reviewed and approved under the protocol number HUT100602MOU by the VA IACUC committee (Subcommittee on Animal Studies, NIH assurance number A3088-01) at the VA Palo Alto Health Care System and in accordance with the PHS Policy on Humane Care and Use of Laboratory Animals.

### Cell culture and mouse model

Control Huh7 cells and Huh7 cells with genome-length replicon, subgenomic replicon, or the tet-inducible core expression construct were used for this study. All three HCV protein-expressing cell lines have been described previously [20], [26]. Genome-length replicon and subgenomic replicon were derived from a genotype 1a H77c infectious molecular clone [20]. The genome-length replicon encodes the full-length HCV polyprotein (core, E1, E2, p7, and NS2-NS5), whereas the subgenomic replicon encodes only the non-structural proteins NS2-NS5B [20]. The tet-inducible core-expressing cells express core protein in the absence of doxycycline. These cells are referred to as Core-on cells when core expression is turned on and as Core-off cells when core expression is turned off by the addition of doxycycline to the culture medium. All cells were maintained in DMEM with 10% tetracycline-free fetal calf serum (FCS, Clontech, Mountain View, CA, USA), Pen/Strep (50 IU/ml penicillin and 50 mg/ml streptomycin), and G418 (200 µg/ml) plus the following antibiotics: genome-length and subgenomic replicon cells, 5 µg/ml blasticidin; Core-off cells, 20 µg/ml doxycycline. When cells were plated for an experiment, only DMEM with 10% tetracycline-free FCS and Pen/Strep was used. All cells were incubated at 37°C with 5% CO_2_. Expression of HCV proteins in genome-length and subgenomic replicon cells were monitored by following the specific activities of the reporter gene, secreted alkaline phosphatase (SEAP), in the culture medium at 48 to 72 hrs after the initial plating [51]; HCV core protein expression in genome-length replicon and Core-on cells were monitored by western blot analysis.

HCV transgenic mice expressing the full-length HCV polyprotein [12] were used at 3 months of age. Only male mice were used in this study. All mice were kept in a barrier facility with a 12-hr dark-light cycle, given food and water *ad libitum*, and maintained in microisolators with a constant temperature between 20°C and 26°C. All animal procedures were reviewed and approved by the IACUC committee at the VA Palo Alto Health Care System and in accordance with the PHS Policy on Humane Care and Use of Laboratory Animals.

### Ultrastructural analysis

Control Huh7 cells and genome-length replicon, subgenomic replicon, Core-on, and Core-off cells were fixed with 2.5% glutaraldehyde in 0.1 M Na Cacodylate, pH 7.4 for 2 hrs, post-fixed with 2% aqueous osmium tetroxide for 2.5 hrs, and subsequently stained *en bloc* in 2.5% uranyl acetate (in water) overnight before dehydration and embedding in Eponate 12 resin (Ted Pella, Inc., Redding, CA, USA). Thick (1 µm) sections of the embedded cells were examined at the light microscopic level. The cell blocks were further trimmed to obtain thin sections (80 nm), stained with saturated solution of uranyl acetate (15 min) followed by Reynolds' lead citrate (8 min), and examined with a JEOL JEM 100CX II transmission electron microscope (JEOL Ltd., Tokyo, Japan).

HCV transgenic and non-transgenic mice were perfused through the left ventricle of the heart, first with 10 U/ml heparin in saline until the liver was cleared of blood (about 5 min), then with fixative (2% glutaraldehyde and 2% paraformaldehyde in 0.1M Na Cacodylate, pH 7.4) until the liver was completely fixed (about 6–8 min). Heparin and fixative solutions were delivered by a peristaltic pump (VWR, Westchester, PA, USA) with the flow rate set at 4.5 ml/min. One mm cubes were prepared from the fixed liver and were left to continue fixation at room temperature (RT) overnight. These specimens were processed and embedded in Eponate 12 resin as described above.

### Measurements of subcellular structures

For mitochondrial size determination, electron micrographs taken at 7.2K magnification were used, and mitochondrial sizes from 3–4 cells from each cell line were measured. Image J was used to determine the area occupied as pixel number, which was then converted to µm^2^. For determination of the number of mitochondria and autophagocytic vacuoles/vesicles, electron micrographs taken at low magnifications (1.9–3.6K), with the requirement of being able to fit an entire cell into the view, were used and 10–26 cells from each cell line were analyzed.

### MitoSOX and MitoTracker staining

For live MitoSOX and MitoTracker staining and imaging, 2×10^4^ cells were seeded onto each chamber of tissue culture treated 8-chamber slides (Millicell EZ slides, Millipore, Billerica, MA, USA) and incubated overnight. For MitoSOX staining to detect superoxide generation in the mitochondria, culture media was replaced with 200 µL of staining solution containing 5 µM MitoSOX Red (Invitrogen, Carlsbad, CA, USA) and 5 µg Hoechst 33342 (for nuclear staining, Invitrogen) in HBSS, and cells were incubated at 37°C for 10 minutes. For equilibration, staining solution was replaced with 500 µL pre-warmed HBSS and returned to 37°C for another 10 minutes before imaging. For MitoTracker staining to visualize the mitochondrial network, culture media was replaced with 100 µL of staining solution containing 200 nM MitoTracker Green FM (Invitrogen) and 5 µg Hoechst 33342 in HBSS, and cells were incubated at 37°C for 30 minutes. The staining solution was then replaced with 500 µL pre-warmed HBSS before imaging. Cells were imaged directly in HBSS with a 40x (NA = 0.6) objective on an Olympus IX71 inverted fluorescence microscope equipped with a Coolsnap HQ monochrome camera (Photometrics, Tucson, AZ, USA). For direct comparison of staining intensity, exposure time was kept constant at 500 msec for MitoSOX and at 200 msec for MitoTracker. Staining intensities of MitoSOX and MitoTracker were determined with Image J as pixel intensities, and a minimum of 40 cells each were analyzed.

### Enzyme activity assays

Expression level of the reporter gene, secreted alkaline phosphatase (SEAP), was used to monitor the expression of the HCV genome-length and subgenomic replicons. Huh7 cells with the genome-length or subgenomic replicon were cultured to ∼80% confluency and cell culture medium from each cell line was removed for SEAP assays. The SEAP reporter assay was performed using the Great EscAPe SEAP Fluorescence Detection Kit (Clontech), and SEAP activities were normalized to total cellular proteins. To determine total aconitase activities, Huh7 and HCV protein-expressing cells were cultured to 90% confluency in 60 mm plates. Cell pellets were resuspended in aconitase buffer (50 mM Tris-Cl, pH 8, 2 mM citric acid, and 0.6 mM MnCl_2_) and passed through two rounds of freeze-thaw cycle between liquid nitrogen and room temperature water to break open membranes. Due to the low level of aconitases in Huh7 cells, total aconitase activities were determined with a kinetic assay [24] coupled to the PMS/MTT color reaction to enhance the sensitivity [25]. Following a 3-minute preincubation at 37°C in the dark, the reaction was continued at 37°C and the OD change was monitored every 5 minutes at 590 nm for up to 60 minutes using a plate reader (SpectraMax M3, Molecular Devices, Mountain View, CA, USA). The reaction appeared to be most linear in the first 30 minutes and consequently, OD change per minute, as a function of enzyme activities, was calculated for that time period. The final results were normalized to the amount of total proteins in the cell lysates.

### Manipulation of redox state

To enhance cellular antioxidant capacity, expression constructs for dual expression of CuZnSOD/cytosolic catalase (SOD1/cCAT) or MnSOD/mitochondrial catalase (SOD2/mCAT) were used (constructed by SKZ, unpublished data). Expression of SOD1 and SOD2 are controlled by elongation factor-1 alpha (EF-1á) promoter, and expression of catalase is controlled by CMV promoter. Cells were grown to 50% confluency in 6-well plates and were transfected with 2.5 µg of each expression construct using *Trans*IT®-LT1 Transfection Reagent (Mirus Bio LLC, Madison, WI, USA). Cell lysates were prepared 36 hrs after the transfection for western blot analysis of LC3. CuZnSOD, MnSOD, and catalase levels were also determined to ensure comparable expression of each protein among different cell lines. To increase oxidative stress, cells were grown to 50% confluency in complete culture medium in 6-well plates and were then switched to 2% FCS-containing medium with 0.25 mM xanthine (X) and 20 mU/ml xanthine oxidase (XO). Since X/XO generates a combination of superoxide and H_2_O_2_, catalase (CAT, 40 mU/ml) was added to a subset of cultures to eliminate H_2_O_2_. Cells were incubated with X/XO or X/XO/CAT for 72 hrs; culture medium was changed every 24 hrs to maintain a steady level of X, XO, and CAT.

### Immunochemistry procedures

Several immunochemical procedures are described below; antibodies used in this study are listed in Table 1.

**Table 1:** Primary and secondary antibodies used in this study.

Target	Origin	Source	Cat no.	Dilution
*Primary antibodies for western blot, in-cell western, and immunocytochemistry*				
Aconitase 1	rabbit polyclonal	Richard Eisenstein	Ref [55]	1∶2,000
Aconitase 2	rabbit polyclonal	Richard Eisenstein	Ref [56]	1∶2,000
â-actin	mouse monoclonal (AC-15)	Sigma	A3854	1∶5,000
â-actin	rabbit polyclonal	Sigma	A5060	1∶2,000
CuZnSOD	rabbit polyclonal	Stressgene	SOD-100	1∶2,000
Cyclin D1	mouse monoclonal (HD-11)	Santa Cruz	SC-246	1∶500
HCV core protein	mouse monoclonal (C7-50)	Affinity Bioreagents	MA1-080	1∶1,000
LC3	rabbit polyclonal	Medical & Biological Laboratories	PM036	1∶1000
MnSOD	rabbit polyclonal	Stressgene	SOD-110	1∶3,000
Peroxiredoxin 1	rabbit polyclonal	Ab Frontier	LF-PA0095	1∶2,000
Peroxiredoxin 3	rabbit polyclonal	Ab Frontier	LF-PA0030	1∶3,000
Peroxiredoxin-SO3	rabbit polyclonal	Abcam	Ab16830	1∶2,000
Thioredoxin 1	rabbit polyclonal	Abcam	Ab16835	1∶2,000
Thioredoxin 2	rabbit polyclonal	Abcam	AB16836	1∶1,000
*Secondary antibodies for western blot, in-cell western, and immunocytochemistry*				
Anti-mouse IgG-IR 680	goat polyclonal	Licor	926–32220	1∶20,000
Anti-mouse IgG-IR 800	goat polyclonal	Licor	926–32210	1∶20,000
Anti-rabbit IgG-Alexa Fluor® 488	goat polyclonal	Invitrogen	A-11008	1∶250
Anti-rabbit IgG-IR 680	goat polyclonal	Licor	926–32221	1∶20,000
Anti-rabbit IgG-IR 800	goat polyclonal	Licor	926–32211	1∶20,000
*Primary antibodies for immunogold EM*				
HCV core protein	mouse monoclonal (C2)	Harry Greenberg	Ref [57]	1∶50 or 1∶20
NS5A	rabbit polyclonal	Licia Tomei	Ref [58]	1∶50
NS5B	rabbit polyclonal	Licia Tomei	Ref [58]	1∶100
*Secondary antibodies for immunogold EM*				
Anti-mouse IgG-15 nm Au	goat polyclonal	Ted-Pella	15752	1∶50
Anti-rabbit IgG-15 nm Au	goat polyclonal	Ted-Pella	15727	1∶50

#### Immunogold labeling

For immunogold EM localization of HCV proteins, cells were fixed (0.1% glutaraldehyde, 4% paraformaldehyde in phosphate buffered saline) and embedded in LR Gold resin (Ted Pella) using techniques described by Berryman and Rodewald [52]. Antibodies were pre-absorbed with Huh7 cell homogenate for 1–24 hrs. Thin sections on grids were blocked with 3% bovine serum albumin in TBS (Tris buffered saline) for 1 hr at RT, incubated with primary antibodies for 1 hr at RT, followed by gold-labeled goat anti-mouse IgG or goat anti-rabbit IgG for 1 hr at RT. The antibody complexes were stabilized with 2% glutaraldehyde in water, and the sections were stained with osmium vapor and lead citrate and examined as described above [53], [54]. Huh7 and Core-off cells were used as negative controls for antibody bindings. Multiple non-overlapping areas were scored for the frequency of HCV antigen localization and for the abundance of the antigen in each area. A positive signal is defined as the presence of at least two gold particles in a given organelle.

#### Immunocytochemistry staining for LC3

To determine the status of LC3 protein, cells were seeded on 12 mm Fisherbrand Coverglass for Growth (Fisher Scientific, Pittsburgh, PA, USA) and cultured in a 24-well plate overnight. Huh7 cells treated overnight with Bafilomycin A1 (200 nM, Wako Chemicals, Richmond, VA, USA) were used as positive controls for autophagy. Cells were fixed in 4% paraformaldehyde (in PBS, pH 8), quenched in 50 mM NH_4_Cl for 10 min, permeabilized in 0.1% Triton X-100/PBS for 10 min, and blocked in 1% BSA for 10 min. Cells were then incubated with 15 µl rabbit anti-LC3 polyclonal antibody for 60 min, followed by goat anti-rabbit IgG conjugated with Alexa Fluor 488 for 30 min. Cell nuclei were stained with 10 µg/ml Hoechst 33258 (Invitrogen). All steps were carried out at RT. Cover slips were washed in water, mounted in a drop of ProLong Gold antifade reagent (Invitrogen), and air-dried in the dark overnight. Cells were imaged with a 40x (NA = 0.75) objective on a Zeiss AxioVision microscope equipped with a Hamamatsu monochrome camera. For direct comparison of staining intensity, exposure time was kept constant at 89 msec.

#### Western blot analyses

To determine the protein level of HCV core protein, CuZn superoxide dismutase (CuZnSOD), Mn superoxide dismutase (MnSOD), Peroxiredoxin 1 and 3 (PRDX1 and 3), sulfonylated peroxiredoxin (PRDX-SO3), and cytosolic and mitochondrial aconitase (ACO1 and ACO2), cell pellets were incubated on ice with Tissue Protein Extraction reagent (T-PER, Fisher Scientific) supplemented with a protease inhibitor cocktail (Roche, Indianapolis, IN, USA), passed through 26 gauge needles several times, and centrifuged at 13,000 g, 4°C, for 2 min to remove cell debris. Total protein concentration was determined with a NanoVue Spectrophotometer (GE Healthcare, Piscataway, NJ, USA), and 50 ìg total proteins per lane were used for western blot analyses. Proteins were separated by 4–20% Mini-PROTEAN® TGX gels (Bio-Rad, Hercules, CA, USA) (core, CuZnSOD, MnSOD, ACO1, and ACO2) or 16% Novex Tris-Glycine gels (Invitrogen) (PRDX1, PRDX3, and PRDX-SO3) and transferred to 0.2 µm nitrocellulose membranes (Bio-Rad). PRDX-SO3 antibody only detects sulfonylated PRDX and is not specific to PRDX1-SO3 or PRDX3-SO3. Consequently, sulfonylated PRDX1 and PRDX3 were distinguished based on their size difference (Figure S5C). To determine LC3 protein levels, cells cultured in 60 mm dishes were washed once with PBS and then scraped directly in the cell lysis buffer (50 mM Tris-Cl, pH 7.6, 150 mM NaCl, 2% SDS, 1 mM EDTA, 1x protease inhibitor cocktail) on ice. To break open the membranes, cell lysates were freeze-thawed two times and passed through a 26 gauge needle five times. Fifty µg of each cell lysate was separated with Any KD Mini-PROTEAN® TGX gels (Bio-Rad) and transferred to 0.2 µm nitrocellulose membranes. The same membranes were used for thioredoxin 1 (TRX1) and thioredoxin 2 (TRX2) analyses. Alternatively, LC3-I and II were separated by 12% NuPAGE Bis-Tris gels (Invitrogen) in a subset of the studies. All membranes were incubated with primary antibodies overnight at 4°C followed by IR-labeled secondary antibodies for 1 hr at RT on a shaker. Membranes were washed three times each between primary and secondary antibody incubation and after secondary antibodies with PBST (PBS with 0.1% Tween 20). Membranes were then analyzed with the Odyssey Infrared Imaging System (Licor Biosciences, Lincoln, NE, USA). Signal intensity of each protein was normalized to that of â-actin. Three to four independent experiments were carried out for each protein; all results are plotted as mean ± SEM.

### Statistical analyses

The statistical analysis program GraphPad Prism (version 4.03, GraphPad Software, Inc., San Diego, CA, USA) was used for data analyses. One-way ANOVA analyses with Dunnett's post-hoc test were carried out initially to see if there was an effect of HCV proteins or treatments on the cellular indices being measured. Two-tailed Student's t tests were then used for pair-wise comparison between Huh7 and Genome-length replicon, subgenomic replicon, or Core-on cells. Student's t tests were also carried out for comparisons between Core-on and Core-off cells.

## Supporting Information

Figure S1 Genome-length replicon, subgenomic replicon, and core protein expression. A, SEAP activities are used to monitor the expression of genome-length and subgenomic replicons in Huh7 cells. B, western blot analysis is used to determine the expression of core protein in genome-length replicon cells and Core-on cells.
(TIF)

Figure S2 Oil-red-O staining for lipid deposit in HCV protein-expressing Huh7 cells. Nuclei are stained with hematoxylin. Pictures were taken using a 20x objective.
(TIF)

Figure S3 Cytosolic aconitase (ACO1) protein levels and total aconitase activities. ACO1 protein levels (A) were determined by western blot analyses, and total aconitase activities (B) were determined with a kinetic assay coupled to the PMS/MTT color reaction. Mitochondrial aconitase (ACO2) protein levels were too low to be reliably quantified. Data are presented as mean ± SEM of three independent experiments.
(TIF)

Figure S4 Overexpression of superoxide dismutase (SOD) and catalase (CAT) in HCV protein-expressing Huh7 cells. Cells were transfected with expression vectors designed for dual expression of CuZnSOD/cytosolic catalase (SOD1/cCAT) or MnSOD/mitochondrial catalase (SOD2/mCAT) to increase antioxidant capacity in the cytosol or mitochondria, respectively. For each set of transfection, the order of cells loaded (from left to right) is Huh7, genome-length, subgenomic, Core-on, and Core-off cells. SOD1 and SOD2 are tagged with V5 and CAT with Myc epitope and are detected with antibodies against these epitopes. Endogenous SOD1, SOD2, and CAT are not detectible by V5 or Myc antibody and are therefore, not visible in this blot.
(TIF)

Figure S5 The redox state of peroxiredoxin 1 (PRDX1) and 3 (PRDX3). The ratios of PRDX1-SO3 to PRDX1 (A) and PRDX3-SO3 to PRDX3 (B) were determined by sequential western blot analyses (see C below). C, representative western blots showing the separation of PRDX1 and PRDX3 in 16% polyacrylamide gels and the sequential binding of specific antibodies to PRDX-SO3, PRDX1, and PRDX3. Data are presented as mean ± SEM of four independent experiments.
(TIF)

Table S1 Immunogold EM localization of HCV proteins. The subcellular location of HCV core, NS5A, and NS5B proteins and the frequency at which they are observed in each organelle by immunogold electron microscopy is presented.
(DOCX)
