# Supplementary figures and images for: Persistent Expression of Hepatitis C Virus Non-Structural Proteins Leads to Increased Autophagy and Mitochondrial Injury in Human Hepatoma Cells
Source: PLoS One. 2011 Dec 2;6(12):e28551. doi: 10.1371/journal.pone.0028551 (PMC3229600; doi:10.1371/journal.pone.0028551)

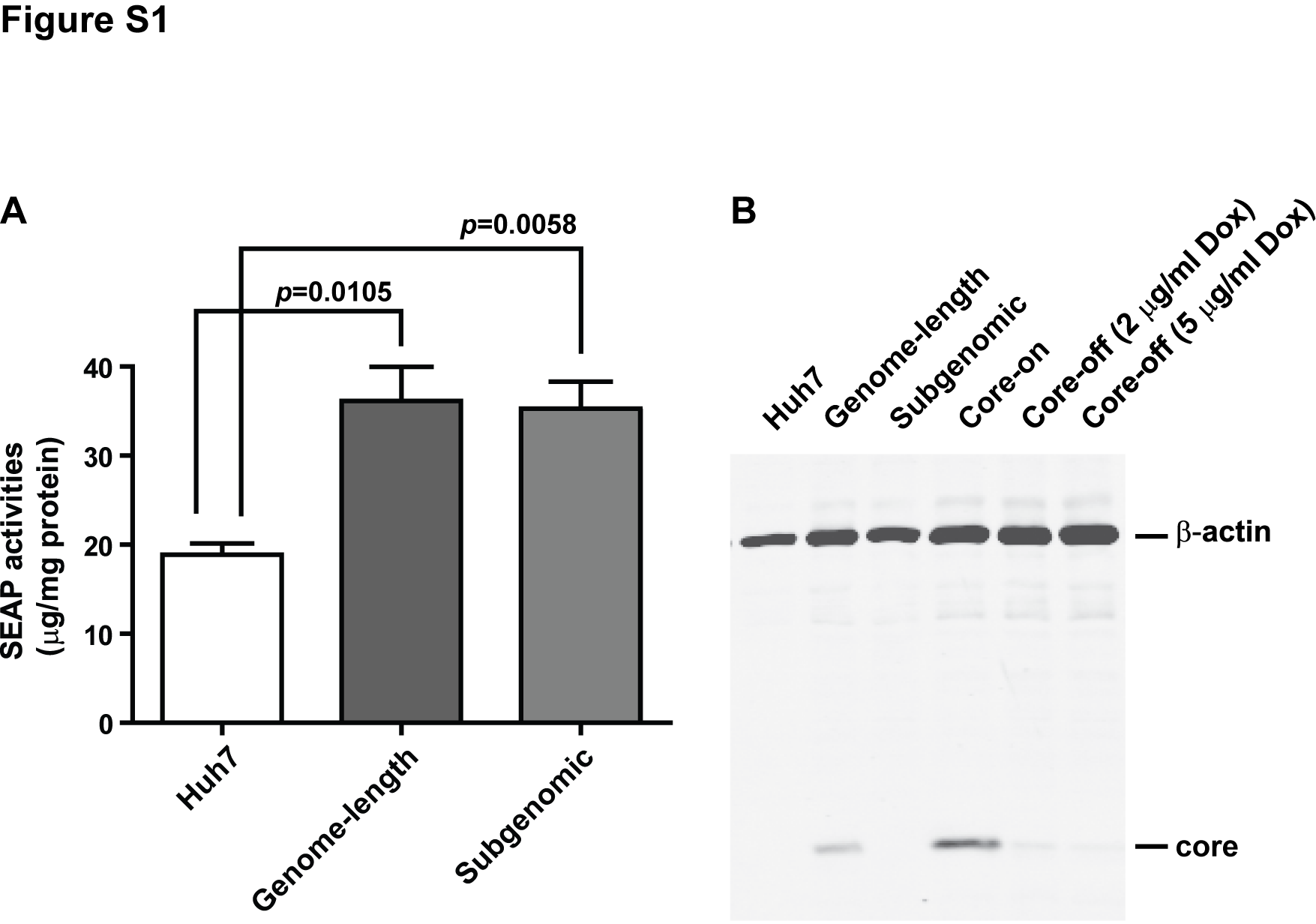

Supplement: Figure S1 — Genome-length replicon, subgenomic replicon, and core protein expression. A, SEAP activities are used to monitor the expression of genome-length and subgenomic replicons in Huh7 cells. B, western blot analysis is used to determine the expression of core protein in genome-length replicon cells and Core-on cells. (TIF) [file pone.0028551.s001.tif]

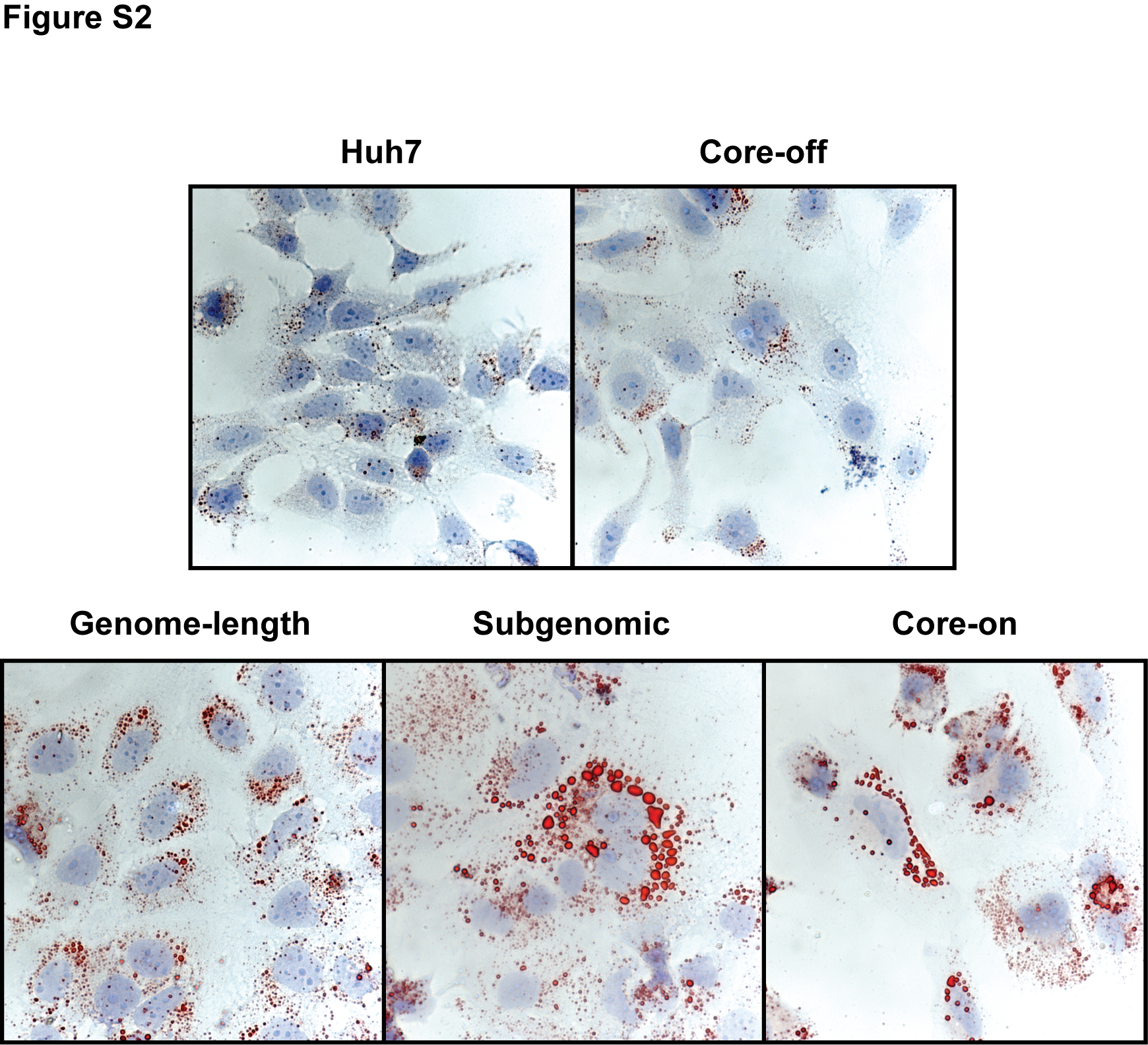

Supplement: Figure S2 — Oil-red-O staining for lipid deposit in HCV protein-expressing Huh7 cells. Nuclei are stained with hematoxylin. Pictures were taken using a 20x objective. (TIF) [file pone.0028551.s002.tif]

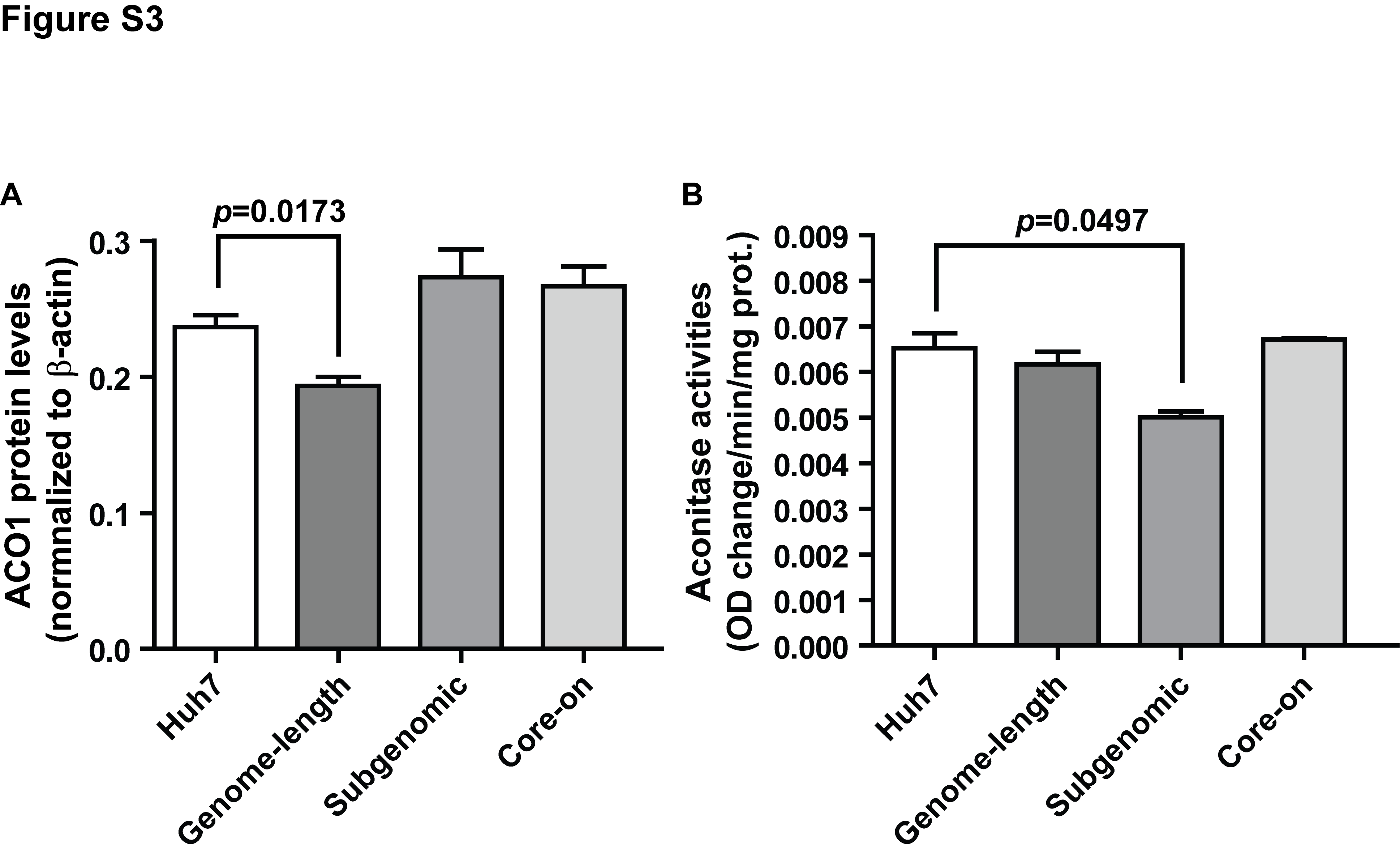

Supplement: Figure S3 — Cytosolic aconitase (ACO1) protein levels and total aconitase activities. ACO1 protein levels (A) were determined by western blot analyses, and total aconitase activities (B) were determined with a kinetic assay coupled to the PMS/MTT color reaction. Mitochondrial aconitase (ACO2) protein levels were too low to be reliably quantified. Data are presented as mean ± SEM of three independent experiments. (TIF) [file pone.0028551.s003.tif]

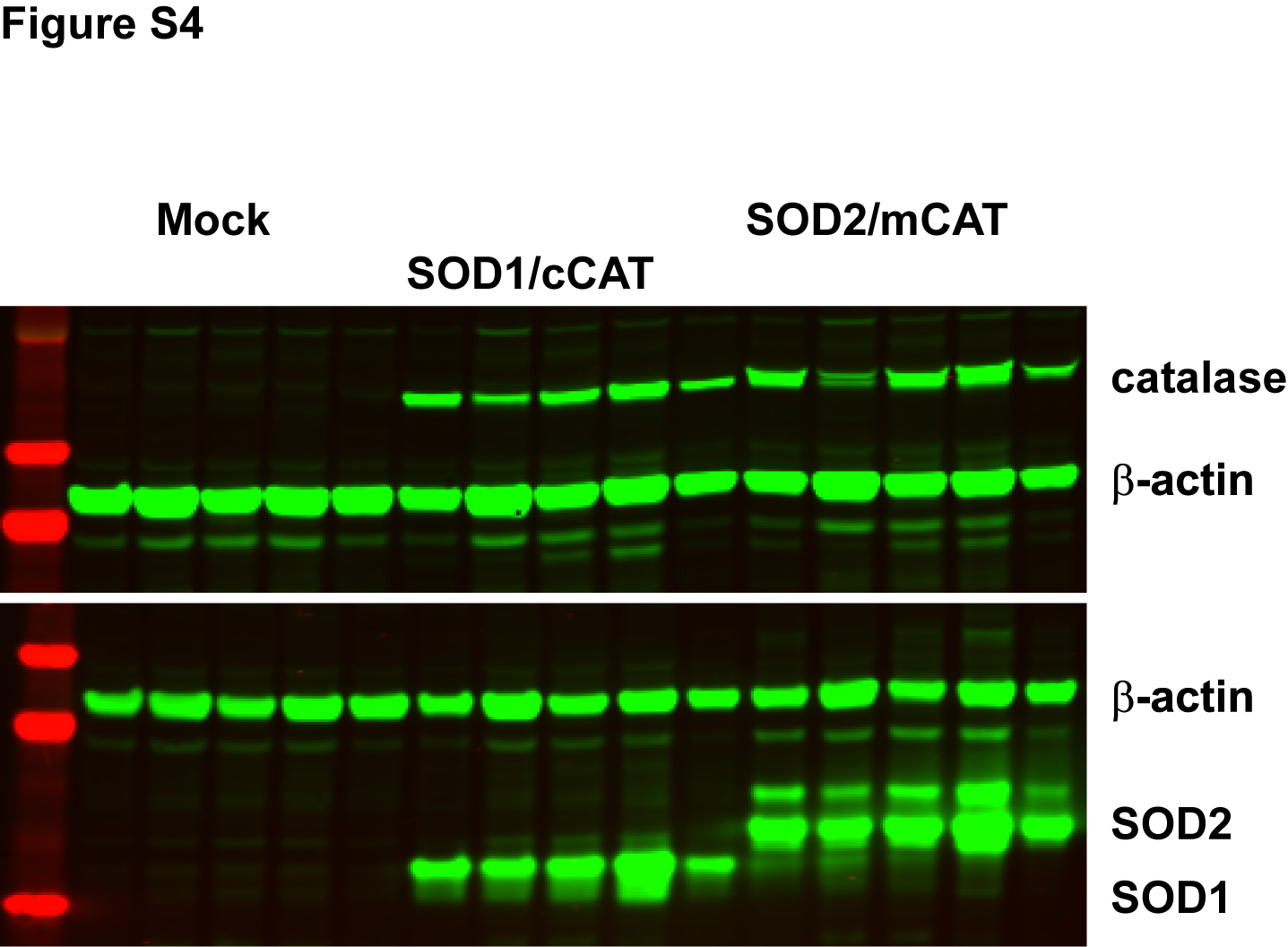

Supplement: Figure S4 — Overexpression of superoxide dismutase (SOD) and catalase (CAT) in HCV protein-expressing Huh7 cells. Cells were transfected with expression vectors designed for dual expression of CuZnSOD/cytosolic catalase (SOD1/cCAT) or MnSOD/mitochondrial catalase (SOD2/mCAT) to increase antioxidant capacity in the cytosol or mitochondria, respectively. For each set of transfection, the order of cells loaded (from left to right) is Huh7, genome-length, subgenomic, Core-on, and Core-off cells. SOD1 and SOD2 are tagged with V5 and CAT with Myc epitope and are detected with antibodies against these epitopes. Endogenous SOD1, SOD2, and CAT are not detectible by V5 or Myc antibody and are therefore, not visible in this blot. (TIF) [file pone.0028551.s004.tif]

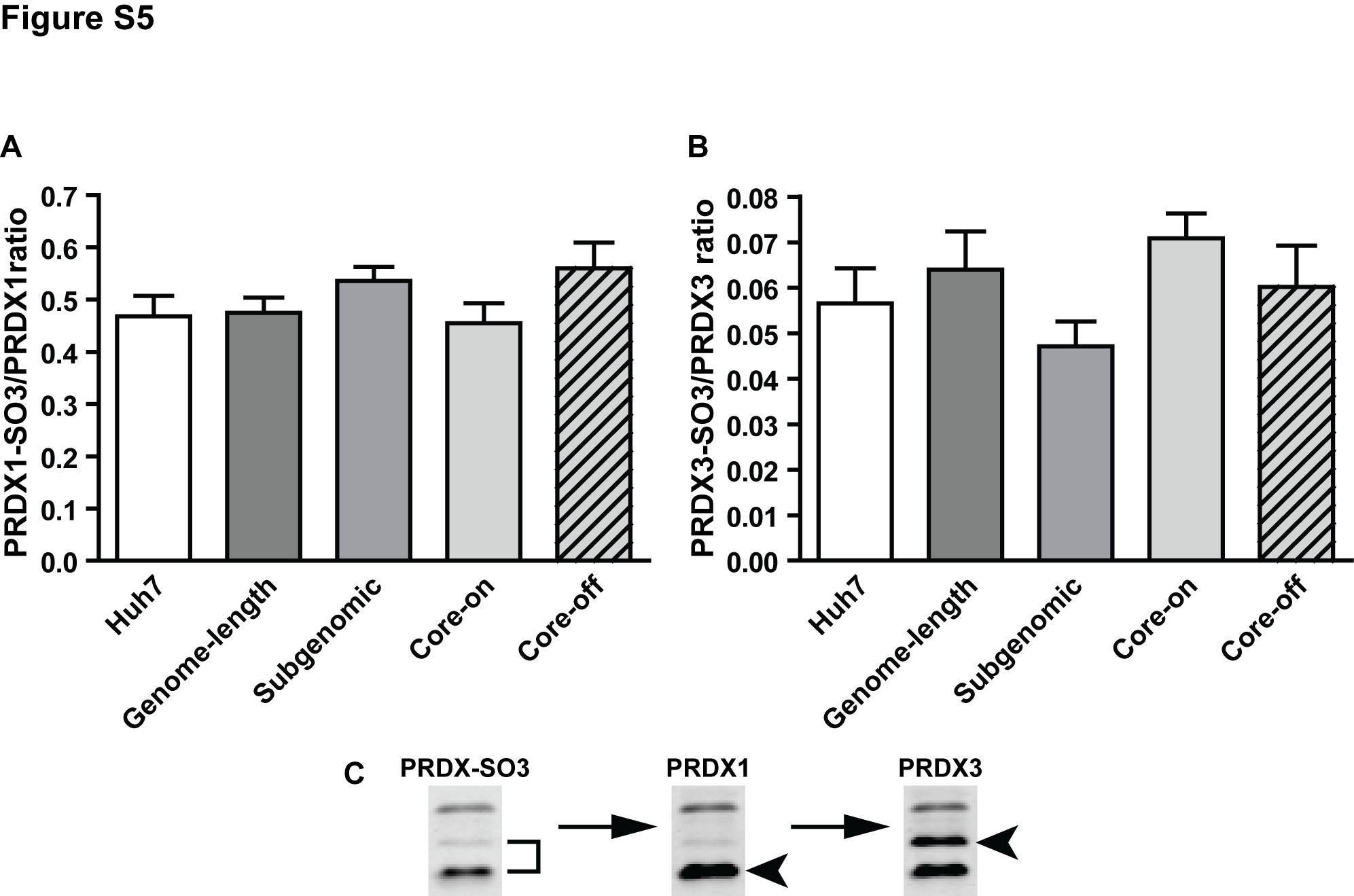

Supplement: Figure S5 — The redox state of peroxiredoxin 1 (PRDX1) and 3 (PRDX3). The ratios of PRDX1-SO3 to PRDX1 (A) and PRDX3-SO3 to PRDX3 (B) were determined by sequential western blot analyses (see C below). C, representative western blots showing the separation of PRDX1 and PRDX3 in 16% polyacrylamide gels and the sequential binding of specific antibodies to PRDX-SO3, PRDX1, and PRDX3. Data are presented as mean ± SEM of four independent experiments. (TIF) [file pone.0028551.s005.tif]
