# Supplementary material for: Persistent Expression of Hepatitis C Virus Non-Structural Proteins Leads to Increased Autophagy and Mitochondrial Injury in Human Hepatoma Cells
Source: PLoS One. 2011 Dec 2;6(12):e28551. doi: 10.1371/journal.pone.0028551 (PMC3229600; doi:10.1371/journal.pone.0028551)
Supplement: Table S1 — Immunogold EM localization of HCV proteins. The subcellular location of HCV core, NS5A, and NS5B proteins and the frequency at which they are observed in each organelle by immunogold electron microscopy is presented. (DOCX) [file pone.0028551.s006.docx]

**Table S1 Immunogold EM localization of HCV proteins**

|  |  | **mitochondria** | | |  |  |  |  |  |  |
| --- | --- | --- | --- | --- | --- | --- | --- | --- | --- | --- |
| **antigen** |  | **outer membrane** | **inner membrane** | **matrix** | **RER** | **SER** | **nucleus** | **lipid** | **Golgi** | **autophagocytic vacuoles** |
| Core (17)^a^ | % positive^b^ | 59% | 35% | 71% | 18% | 47% | 12% | 6% | 35% | 12% |
|  | avg signal intensity^c^ | + | ++ | ++ | ++ | + | + | + | + | + |
| NS5A (19)^a^ | % positive^b^ | 79% | 58% | 68% | 47% | 21% | 32% | 32% | 16% | 26% |
|  | avg signal intensity^c^ | ++ | ++ | ++ | ++ | + | ++ | ++ | ++ | ++ |
| NS5B (27)^a^ | % positive^b^ | 33% | 48% | 85% | 33% | 4% | 30% | 15% | 22% | 7% |
|  | avg signal intensity^c^ | ++ | ++ | ++ | ++ | + | ++ | ++ | + | +++ |

^a^ Number of electron micrographs examined for subcellular localization.

^b^ Percentage of electron micrographs with at least two gold particles binding to a single organelle or a sub-mitochondrial location.

^c^ + = two gold particles; ++ = 3-6 gold particles; +++ = more than 6 gold particles in a single organelle or a sub-mitochondrial location.
